# Whole genome analysis reveals the genomic complexity in metastatic cutaneous squamous cell carcinoma

**DOI:** 10.3389/fonc.2022.919118

**Published:** 2022-08-02

**Authors:** Amarinder Singh Thind, Bruce Ashford, Dario Strbenac, Jenny Mitchell, Jenny Lee, Simon A. Mueller, Elahe Minaei, Jay R. Perry, Sydney Ch’ng, N. Gopalakrishna Iyer, Jonathan R. Clark, Ruta Gupta, Marie Ranson

**Affiliations:** ^1^ School of Medicine, University of Wollongong, Wollongong, NSW, Australia; ^2^ Illawarra Health and Medical Research Institute, Wollongong, NSW, Australia; ^3^ Illawarra Shoalhaven Local Health District, Wollongong, NSW, Australia; ^4^ Sydney Medical School, Faculty of Medicine and Health, The University of Sydney, NSW, Australia; ^5^ Sydney Head and Neck Cancer Institute, Chris O’Brien Lifehouse, Sydney, NSW, Australia; ^6^ Department of Clinical Medicine, Macquarie University, Sydney, NSW, Australia; ^7^ Department of Otorhinolaryngology, Head and Neck Surgery, Zurich University Hospital and University of Zurich, Zurich, Switzerland; ^8^ School of Chemistry and Molecular Bioscience, University of Wollongong, Wollongong, NSW, Australia; ^9^ Department of Head and Neck Surgery, National Cancer Center, Singapore, Singapore; ^10^ Duke-NUS Medical School, Singapore, Singapore; ^11^ Royal Prince Alfred Institute of Academic Surgery, Sydney Local Health District, Sydney, NSW, Australia; ^12^ Anatomical Pathology, Royal Prince Alfred Hospital, Sydney, NSW, Australia

**Keywords:** CSCC, cutaneous, squamous cell carcinoma, metastases, UTR - Untranslated regions, noncoding, mutations, whole genome sequencing

## Abstract

Metastatic cutaneous squamous cell carcinoma (CSCC) is a highly morbid disease requiring radical surgery and adjuvant therapy, which is associated with a poor prognosis. Yet, compared to other advanced malignancies, relatively little is known of the genomic landscape of metastatic CSCC. We have previously reported the mutational signatures and mutational patterns of CCCTC-binding factor (CTCF) regions in metastatic CSCC. However, many other genomic components (indel signatures, non-coding drivers, and structural variants) of metastatic CSCC have not been reported. To this end, we performed whole genome sequencing on lymph node metastases and blood DNA from 25 CSCC patients with regional metastases of the head and neck. We designed a multifaceted computational analysis at the whole genome level to provide a more comprehensive perspective of the genomic landscape of metastatic CSCC. In the non-coding genome, 3′ untranslated region (3′UTR) regions of *EVC* (48% of specimens), *PPP1R1A* (48% of specimens), and *ABCA4* (20% of specimens) along with the tumor-suppressing long non-coding RNA (lncRNA) LINC01003 (64% of specimens) were significantly functionally altered (Q-value < 0.05) and represent potential non-coding biomarkers of CSCC. Recurrent copy number loss in the tumor suppressor gene *PTPRD* was observed. Gene amplification was much less frequent, and few genes were recurrently amplified. Single nucleotide variants driver analyses from three tools confirmed *TP53* and *CDKN2A* as recurrently mutated genes but also identified *C9* as a potential novel driver in this disease. Furthermore, indel signature analysis highlighted the dominance of ID signature 13 (ID13) followed by ID8 and ID9. ID9 has previously been shown to have no association with skin melanoma, unlike ID13 and ID8, suggesting a novel pattern of indel variation in metastatic CSCC. The enrichment analysis of various genetically altered candidates shows enrichment of “TGF-beta regulation of extracellular matrix” and “cell cycle G1 to S check points.” These enriched terms are associated with genetic instability, cell proliferation, and migration as mechanisms of genomic drivers of metastatic CSCC.

## Introduction

Cutaneous squamous cell carcinoma (CSCC) is the second most common malignancy, after basal cell carcinoma (BCC), affecting up to 1,000,000 people in the United States annually (1). In time, and as a result of the aging population and changing ratios of BCC/CSCC, the mortality rate of CSCC is likely to exceed that of melanoma (2). Although primary CSCC is common, metastasis only occurs in 2%–5% of CSCC (3–5). CSCCs arising in the head and neck generally show a predictable pattern of spread, predominantly metastasizing to the intraparotid, level II (upper jugular), and perifacial lymph nodes (4). CSCCs that have metastasized to regional lymph nodes are associated with a worse prognosis (6), with modest progress made in the management of regionally advanced disease over the last 15 years. Most patients with regional metastases from CSCC of the head and neck are managed with a multimodality approach, which usually involves surgery (parotidectomy and neck dissection) and adjuvant external beam radiotherapy depending on the site and stage at the time of diagnosis (7–9). More recently immunotherapy has attracted great interest as a potential alternative for unresectable or distant metastatic disease (10, 11).

Despite the very high incidence, relatively little is known regarding the genomic landscape of metastatic CSCC. We have previously described the genomic mutational burden, mutational signatures, and mutations in CCCTC-binding factor regions using whole genome sequencing (WGS) data from 15 CSCC metastases (12) and associated cell lines (13). However, the majority of studies to date has reported on somatic variation in primary CSCC (14–17) and/or CSCC metastases (17–21), using whole exome sequencing (WES) and/or targeted next generation sequencing, which by definition focuses on the coding genome. Thus, the extent of analysis of non-coding (including regulatory) regions of the genome is limited and varies across studies. Pickering et al. (21), the only study employing WES and incorporating 32 primary and only seven metastatic samples, did not include regulatory or non-exome regions analysis. Both Li et al. (19) [29 lymph node metastatic formalin fixed paraffin embedded (FFPE) samples] and Zehir et al. (18) (MSK-IMPACT) (28 primary and 27 metastatic FFPE samples) used targeted next-generation sequencing (NGS), with limited non-coding analysis. Zehir et al. (18) specifically included the *TERT* promoter in their targeted panels but otherwise included no regulatory elements. Li et al. (19) similarly did not include regulatory or non-coding variant analysis. Yilmaz et al. (17) performed WES and/or targeted NGS on 18 metastatic and 10 primary FFPE CSCC samples and reported coding gene drivers based purely on mutational frequencies, without adjusting for gene length or covariates. Additional functional driver predictions analysis would be required to confidently call genes as drivers (22). Furthermore, FFPE processing has well-known impacts on the quality of DNA for sequencing analyses (23), and it is important to note that for most of the metastatic studies, FFPE samples were collected. Furthermore, none of these studies addressed variation in either 5′ or 3′ untranslated regions (UTRs) or other non-coding elements such as promoters (other than *TERT* promoter) or long non-coding RNAs (lncRNAs). Sequence variants occurring within these functional non-coding elements are important, as they have the potential to alter gene expression. For example, lncRNAs are thought to influence the expression of proteins by pre- and post-translational influences on DNA/RNA and proteins, chromatin function, miRNA activity, and signaling pathways by an array of mechanisms (24, 25). 3′UTRs regulate crucial aspects of post-transcriptional gene regulation (26). Mutations in these regions can deregulate gene expression by disrupting miRNA–mRNA interactions, in which both tumor suppressor genes and oncogenes can drive cancer progression (27, 28). This variation in the so-called *cis-elements* can also impact gene expression by altering translation initiation in cancer (29).

Given the shortcomings associated with WES and NGS analyses of complex genomes, in the current report we have performed WGS on 25 metastatic CSCC samples and applied a detailed, multifaceted computational analysis at the whole genome level to provide a comprehensive understanding of the genomic landscape of metastatic CSCC. This included processing of WGS data for somatic variations in both coding and non-coding regions and indel signatures, apart from structural variants and copy number alterations analyses. For non-coding genomic regions, we have focused on UTRs, lncRNA, and promoter regions, as these represent non-coding regions that are most accessible to interrogation in high mutational burden tumors using currently available tools.

## Materials and methods

### Study population, sample collection, and processing

This study was undertaken with Institutional Human Research Ethics approval (UOW/ISLHD HREC14/397). Thirty-two patients with resectable metastatic CSCC were identified by the treating surgeons preoperatively. Clinicopathological data including age, sex, extent of nodal metastases, histology, and immunosuppression status were collected. In addition to whole blood (for germline DNA), sections of fresh tumor from nodal metastases were collected during surgery and immediately snap frozen. These sections were used for DNA extraction (Qiagen AllPrep, Qiagen, Hilden, Germany) and for cellularity estimates. Only samples with >30% tumor (range, 35%–95%) proceeded to DNA quality control (QC). QC comprised spectrophotometry (Nanodrop 2000 Thermo Fisher Scientific Inc.), gel electrophoresis, and single nucleotide polymorphism (SNP) array. Of the 32 samples sequenced, 25 passed QC (96% from men) (**Table 1**). The remaining seven samples had insufficient clonal tumor content [median variant reads ≤ 5 or median variant allele frequency (VAF) < 0.1] or had an extreme GC bias as determined by PURity and PLoidy Estimator (PURPLE) (30). Briefly, if more than 220 copy number segments were unsupported by a corresponding structural variants at either end, the sample was flagged as fail-segment. The mean sequencing coverage of the 25 samples was 94.56× (range, 64–143) for tumor and 41.08× (range, 30–56) for blood.

**Table 1:** Clinicopathological data of the cohort of 25 patients with CSCC lymph node metastases.

Sample	Age (years)	Sex	Primary location	Metastasis location	Nodal stage^1^	Lymph node ratio^2^	Extracapsular spread	Grade^3^	Immuno-suppressive treatment
CSCC_0001	30	male	left lip	left neck	N3b	3/27	yes	1	no
CSCC_0002	78	male	right ear	right parotid	N3b	2/52	yes	3	no
CSCC_0003	74	male	unknown	right parotid	N3b	2/42	yes	3	no
CSCC_0004	64	male	bilateral lip	bilateral neck	N2c	3/55	no	2	no
CSCC_0005	78	male	left forehead	left parotid	N2a	4/4	Not stated	3	no
CSCC_0006	69	male	left cheek	left neck	N3b	2/42	yes	3	azathioprine
CSCC_0007	87	male	unknown	left neck	N2b	1/16	no	3	no
CSCC_0009	66	male	bilateral forehead	right neck	N3b	3/109	yes	2	cyclosporine A, tacrolimus
CSCC_0010	64	male	left scalp	left neck	N3b	2/11	yes	3	no
CSCC_0011	69	male	unknown	right parotid	N3b	3/108	yes	3	no
CSCC_0012	77	male	right nose	right neck	N3b	4/64	yes	2	no
CSCC_0013	77	male	right pinna	right parotid	N3b	1/1	yes	2	no
CSCC_0014	79	female	left cheek	left perifacial	N3b	1/1	yes	3	no
CSCC_0022	66	male	scalp	left neck	N3b	3/24	yes	3	no
CSCC_0024	54	male	lip	right neck	N3b	3/32	yes	2	no
CSCC_0025	82	male	parotid	Parotid	N1	1/15	no	3	no
CSCC_0066	56	male	Unknown	Parotid	N1	1/1	no	3	no
CSCC_0124	80	male	Parotid	Parotid	N3b	1/6	yes	Not stated	no
CSCC_0125	43	male	parotid	parotid	N3b	1/20	not stated	not stated	no
CSCC_0126	66	male	left temple	left neck	N3b	3/8	yes	3	no
CSCC_0130	70	male	unknown	left parotid	N3b	1/6	yes	3	no
CSCC_0132	76	male	right ear	parotid/neck	N2b	23/43	no	3	no
CSCC_0133	75	male	unknown	parotid	N3b	1/4	yes	not stated	no
CSCC_0134	71	male	unknown	right neck	N3b	9/17	yes	not stated	no
CSCC_0135	82	male	unknown	right neck	3b	1/48	yes	3	no

^1^Staging according to AJCC 8th edition.

^2^Lymph node ratio (Number of positive nodes/total nodes harvested).

^3^Grade 1: well differentiated; Grade 2: moderately differentiated; Grade 3: poorly differentiated.

### Variant calling and functional significance of SNVs and indels

FASTQ reads were aligned to reference genome GRChr38 using BWA-kit version 0.7.17 (BWA-MEM read aligner) (for details, refer to https://github.com/Sydney-Informatics-Hub/Fastq-to-BAM). The Genome Analysis Tool Kit (GATK) 4.1.2.0 and its BaseRecalibrator tool was used to refine the read alignment. SNPs and insertion–deletion (indel) variants were called by implementing GATK’s Best Practices Workflow. These pipelines use HaplotypeCaller for germline short variant discovery and Mutech2 caller for somatic short variant discovery for SNVs and indels (for details, refer to https://github.com/Sydney-Informatics-Hub/Somatic-ShortV). Furthermore, variants effect prediction and annotations were completed using OpenCravat platform (31). Mutation Annotation Format (MAF) files were generated based on variant effect predictor annotations. Three different methods for driver discovery were then used; OncodriveFML (32), MutSigCV (22), and dNdScv (33).

OncodriveFML predicts the functional significance of both coding and non-coding variants, as it is one of the few tools designed for non-coding genomic analysis (32). It first determines the functional impact of the observed somatic mutations using Combined Annotation Dependent Depletion (CADD) for specified genomic elements (UTR, promotor, and coding regions) across the cohort. Later, for the statistical significance, it compares the average functional impact score of the observed mutations in the element with the average functional impact scores of a similar number of the random mutational set. The CADD score provides a priority for identifying mutations with functional, deleterious, and pathogenic impacts. These scores are calculated by combining the information from multiple annotations into a single metric.

MutSigCV identifies genes that are mutated more often than expected by chance and reduces the number of false positives in the generated list of significant genes, which is especially useful for tumors, such as metastatic CSCC, with high mutation rates (22). This is achieved by incorporating various types of information such as patient-specific mutation frequencies and mutation spectra, gene-specific mutation rates, expression levels, and replication times.

dNdScv is designed to test for positive and negative selection in cancer genomes (33). As UV-induced cancer genomes such as CSCC can affect the accuracy of the dNdScv model, we carefully monitored the annotation of CC>TT changes (sometimes reported as C>T changes). Results report significance for missense and truncating mutations and indels as global p-values. Genes that were falsely flagged as significant with negative selection were not considered for this analysis.

For downstream analysis, genes that were predicted to be driver genes by at least two of these tools were considered. First, genes with significant p-values <0.005 were filtered from each of the three tools, and shared genes were determined using a Venn diagram. We then compared the functional impact of SNVs in these selected driver genes to previously reported primary and metastatic CSCC data (18, 19, 21, 34) available on cBioportal (35). This included 92 samples of metastatic CSCC (WES= 10, targeted NGS = 82) and 88 samples of primary CSCC (WES=32, targeted NGS=56).

### Copy number variation

Copy number alterations in the 25 metastatic genomes were derived using PURPLE (30), which estimates copy number and purity of tumor sample by using read depth ratio from COBALT and tumor B-allele frequency (BAF) from AMBER. The pipeline is available at github of HMF Tools (https://github.com/hartwigmedical/hmftools). Driver genes with significant amplifications and deletions were then identified using PURPLE driver copy number outputs. For driver genes, PURPLE searches for genes with high level amplification (minimum exonic copy number > 3 * sample ploidy) and deletion (minimum exonic copy number < 0.5) and then uses iteration to establish the most significant focal peaks.

GRIDSS2 and its companion interpreter tool LINX were employed for somatic structural variant analysis and gene fusion (36). COSMIC3-based SNVs and indels signatures from the whole genome were built using MutationalPatterns (37) software.

The driver gene candidates obtained from various genetic alteration analyses such as copy number variation drivers, somatic variant drivers, and other non-coding drivers were combined for enrichment analysis. In the case of copy number gain/loss, we selected only those genes affected in >20% of the samples in our cohort. Using the Enrichr web application (38), we determined the involvement of the candidate driver genes in various cellular components of the cells, biological pathways, and predicted miRNA and drug targets.

## Results

### Patient characteristics and clinicopathological data

Twenty-five metastatic CSCC samples from lymph nodes in the head and neck region were collected between 2015 and 2019 that passed WGS QC criteria for analysis (**Table 1**). The median age of patients was 69 (range, 30–87), and 24/25 (96%) were male. While this sex disparity is a limitation of our study in that potential sex differences may have been missed, it is in keeping with the disease burden seen in our practice in NSW, Australia, particularly for advanced and metastatic CSCC (39). This is in keeping with findings that age, male sex, and immunosuppression are among the risk factors for metastasis (40). Two patients were immunocompromised; one patient was on long-term azathioprine for rheumatoid arthritis, and the other was on a combination of cyclophosphamide and tacrolimus following solid organ transplantation.

The location of the index primary lesion was known in 11 patients (**Table 1**). Nodal metastases were isolated from the neck in 13 patients and in the parotid in 12 patients. The majority of patients had either moderately differentiated (n = 8) or poorly differentiated (n = 12) CSCC, with evidence of extranodal extension found in 20/25 (80%) nodal samples.

### Tumor mutational burden

**Figure 1:**
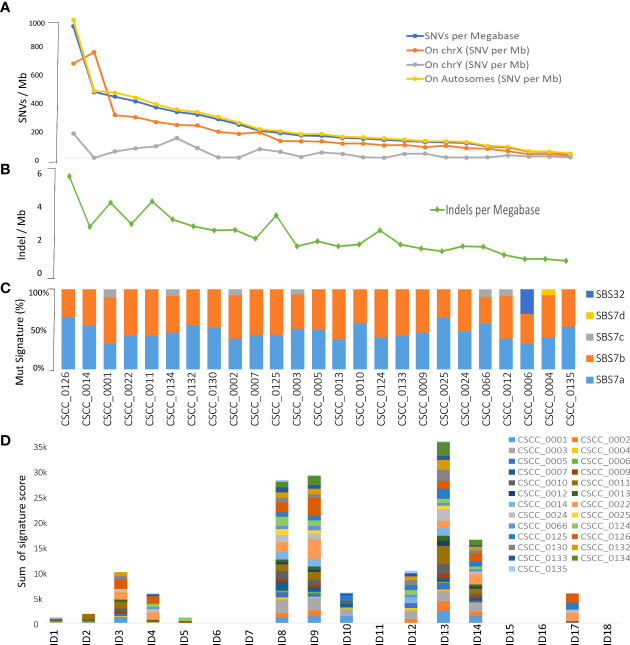
Overview of tumor mutational burden and signatures (whole genome-based). Panels **(A, B)** illustrate the indel and SNV mutational burden in each sample, respectively. Panels **(C, D)** show indel (ID) and SNV mutational signatures for each sample, respectively, obtained using COSMIC V3.2 database. Full details are available in **Supplementary Table S2**.

Based on whole genome level calculations, the average tumor mutational burden (TMB) for SNVs and indels across the 25 cases was 238.7 mutations per megabase (median, 166.99 mutations/Mb; range, 32.52–995.66 mutations/Mb) and 2.25 indel/megabase (range, 0.63–5.9 mutations/Mb), respectively (**Figures 1A, B**; **Supplementary Table S1**) with the majority of somatic variants occurring in the non-coding regions as expected (12). The only female tumor in this cohort had the second highest TMB at 499 mutations/Mb. There was no correlation between age, differentiation, nodal stage, or extracapsular spread of the metastasis and TMB.

### Mutational signatures

We performed mutational signature analyses of the 25 genomes based on COSMIC V.3.2 (https://cancer.sanger.ac.uk/signatures/). Signatures are designated as single base substitution (SBS) or small insertion and deletion (ID) signatures. SBS signatures 7a and 7b were the most prevalent (**Figure 1C**; **Supplementary Table S2**) in keeping with a UV association in metastatic CSCC as we previously reported in a smaller cohort using COSMIC V2 (12). Substantial representation of SBS7c was also seen. SBS32 and SBS7d were observed in one sample. Indel signature analysis showed that ID8, 9, and 13 dominated over others (**Figure 1D**; **Supplementary Table S2**).

### Short variants

#### Coding short variants

**Figure 2:**
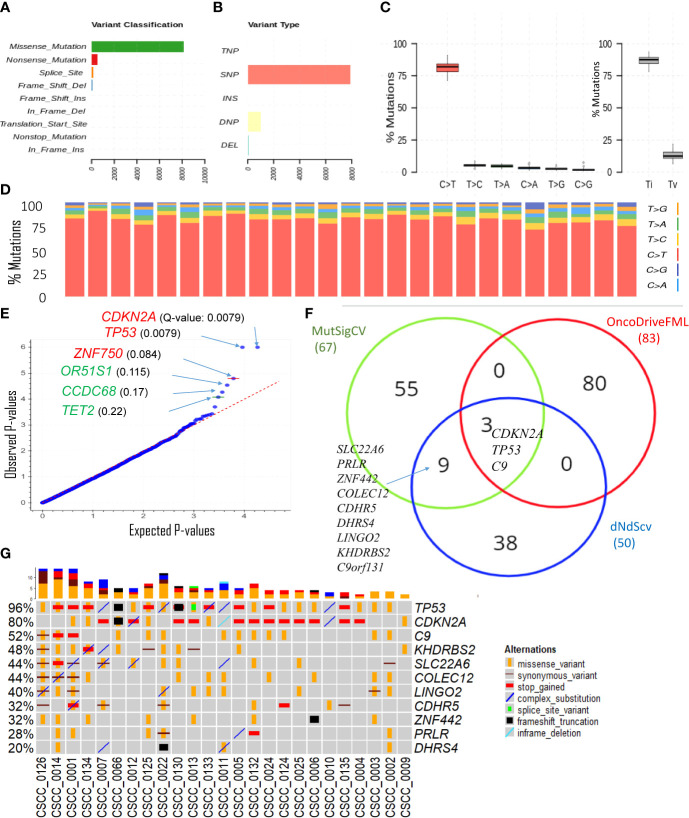
Overview of key coding mutations. **(A)** Variants classification, **(B)** variant types, where SNP, DNP, TNP, INS, and DEL are single nucleotide polymorphisms, double nucleotide polymorphisms, triple nucleotide polymorphisms, insertion, and deletion, respectively (**C**, left panel) % of various transitions, (**C**, right panel) Ti (transition) and Tv (transversion) in all 25 samples, and **(D)** % transitions for each sample. **(E)** Driver coding genes prediction results from OncodriveFML tool. The plot shows the most significantly altered genes (in the plots above the red line, Q-values are below 0.1). Q-values are corrected p-values using the Benjamini/Hochberg correction. **(F)** Venn diagram showing the overlap of genes predicted to be driver genes (p-value < 0.005) by three different driver detection tools, i.e., OncoDriveFML, MutSigCV, and dNdScv. (For details, refer to **Supplementary Table S3**). For further analysis, genes predicted to be driver genes by at least two tools were considered. **(G)** Detailed sample-level information of the SNVs and types of variants in the top altered genes (mentioned in **Figure 2**).

The overwhelming majority of coding SNVs were missense mutations, followed by nonsense mutation, which represented <5% of variants (**Figure 2A**). **Figure 2B** shows various DNA sequence alterations, including single, double, and triple nucleotide variants and insertion and deletion (**Supplementary Data 1**). Over 80% of SNVs were C>T (**Figures 2C, D**). This is consistent with the dominant effect of UV radiation on pyrimidine bases and the UV signature referred to above and is independent of the degree of differentiation or any other clinicopathological feature. Genes predicted to be driver genes *via* OncoDriveFML include *TP53*, *CDKN2A*, and *ZNF730* having Q-values <0.1 (**Figure 2E**). MutSigCV and dNdScv analyses also found *TP53* and *CDKN2A* as the most significant mutated driver genes in our cohort (**Supplementary Table S3**). Genes that were predicted to be driver genes (p-value < 0.005) by at least two tools were considered for downstream analyses (**Figure 2F**). This resulted in 12 genes: *TP53*, *CDKN2A*, *C9*, *C9orf131*, *SLC22A6*, *KHDRBS2*, *COLEC12*, *LINGO2*, *CDHR5*, *ZNF442*, *PRLR*, and *DHRS4*. Of this list, *TP53*, *CDKN2A*, and *C9* were shared as significant by all three tools. Interrogation of the cBioPortal dataset for CSCC (metastatic = 92 and primary=88 cases) (18, 19, 21) with short variant analysis (**Supplementary Figure S1**) revealed recurrent mutations not only in *TP53* and *CDKN2A* but also in *C9*, *COLEC12*, and *SLC22A6.* Not all genes identified as high impact and recurrent variants in our cohort were included in these targeted studies, which underscores the deficiencies of panel-based analyses in discovery projects.

The only sample with no mutation in *TP53* was CSCC_0009 (**Figure 2G**). The TMB of this sample was 122/Mb or 51% of the average across the cohort. Five samples without *CDKN2A* mutations averaged a TMB of 470/Mb or 201% of the average for the cohort.

#### Variation in non-coding regulatory regions

**Figure 3:**
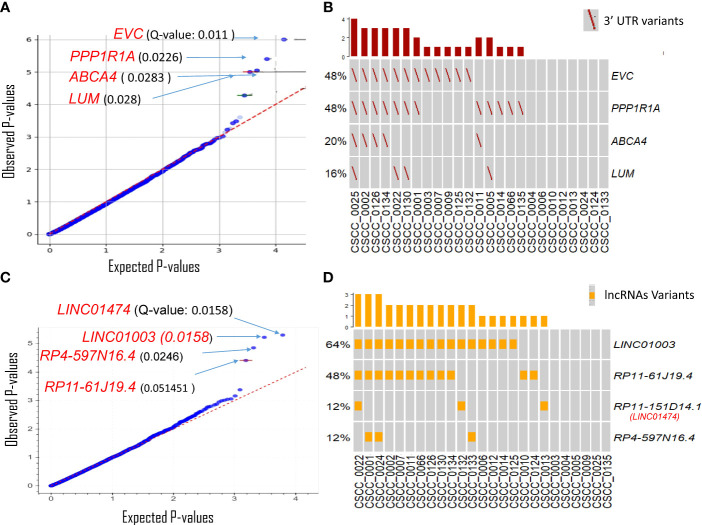
Driver genes prediction in non-coding genomic regions. Plots show the result of OncodrivFML (2.2.0) tool and mutations in the most significantly altered non-coding genes or regions in the cohort of 25 patient samples. **(A)** Potential 3′UTR regions associated driver candidates. **(B)** Variants with significantly altered 3′UTR regions. **(C)** Potential lncRNA driver candidates. **(D)** Variants with significantly altered lncRNAs. Plots in panels **(A)** and **(C)** show the frequency of observed mutations with respect to the expected frequency of the mutations in the corresponding regions. Q-values are corrected p-values using the Benjamini/Hochberg correction. The plots in panels **(B, D)** show frequencies of 3′UTR and lncRNAs variants among the cohorts, respectively.

The 3′UTRs that potentially play an important role in metastatic CSCC were discovered using OncodriveFML. SNVs within the 3′UTR region of *EVC*, *PPP1R1A*, *ABCA4*, and *LUM* showed significantly higher observed functional impact than the expected functional impact (Q-value <0.03) (**Figure 3A**; **Supplementary Table S3**). We observed variation within the 3′UTR of both *EVC* and *PPP1R1A* in 48% of samples with a Q-value of 0.011 and 0.022, respectively (**Figure 3B**; **Supplementary Table S4**). The unique *PPP1R1A* variant with cDNA change of c.*491C>T [Chr12:54579896 (G to A)] was found in five samples (**Supplementary Figure S2**).

There are many reported limitations in the analysis and interpretation of 5′UTRs and promoters for high mutational burden tumors (41–43), a finding that we also observed (**Supplementary Figure 3**). Currently, no robust methodology exists to analyze these regions with confidence in CSCC; thus, analyses of 5′UTRs and promoter regions were not investigated further.

lncRNAs likely to have a potential impact on tumorigenesis were also predicted using OncodriveFML. Four lncRNAs were significantly (q < 0.05) biased towards high-impact mutations i.e., *LINC01474* and *LINC01003*, *RP4-597N16.4*, and *RP11-61J19.4* (**Figure 3C**; **Supplementary Table S3**). Among these, *LINC01474* and *LINC01003* showed a high statistical significance Q-value of 0.0158. lncRNA *LINC01003* was altered in 64% of the cohort. Another recurrently mutated lncRNA in our cohort was *RP11-61J19.4* (48% of samples) (**Figure 3D**; **Supplementary Table S4**).

### Structural and copy number variation

**Figure 4:**
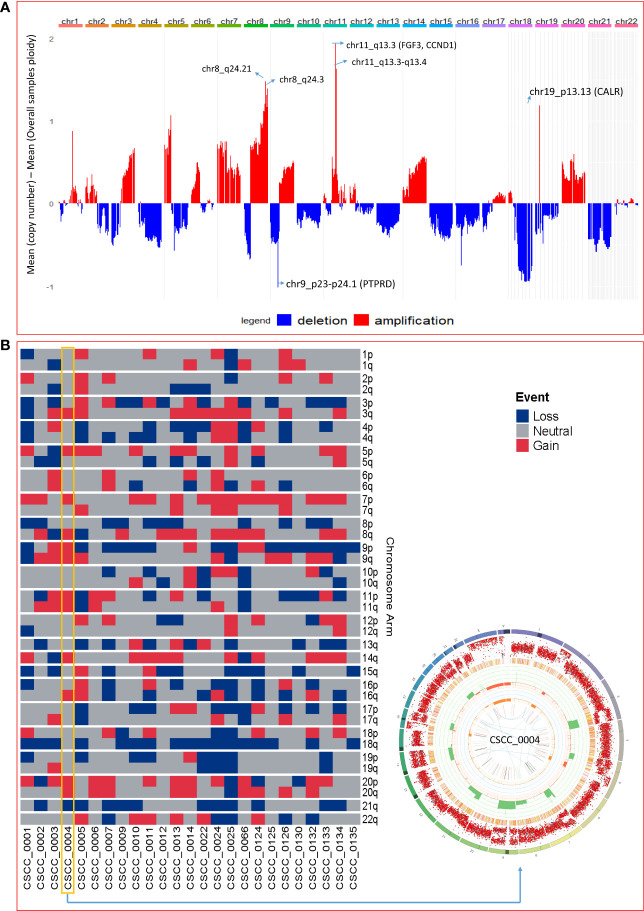
Chromosomal and recurrent genetic copy number variation. **(A)** Combined chromosomal CNV across 25 metastatic CSCC samples at the chromosomal level. The X-axis represents the differences of mean minimum copy number (bands) and means of overall samples ploidy (after adjustment for purity). Refer to **Supplementary Table S5**. **(B)** Chromosomes arm loss and gain at the sample level (red denotes a gain, and blue denotes a loss). Both arms of chromosomes 7 and 5p show gains. 8p, 18q, and 21q show loss. (A chromosome arm is defined to be deleted if at least half of its bases are one or more copies less than the sample ploidy. A chromosome arm is defined to be amplified if at least half of its bases are one or more copies more than the sample ploidy.). Also shown is a Circos plot obtained from the PURPLE pipeline for CSCC_0004 as a representative example that summarizes various information at the sample level. (More details of interpretation at https://github.com/hartwigmedical/hmftools/blob/master/purple/README.md#circos).

The extent of chromosomal copy number gain and loss was averaged across the genome for all 25 tumor samples (**Figure 4A**; **Supplementary Table S5**). Chr5p and 8q were the most frequently amplified regions, with 18q being the region with the most recurrent deletion. At sample level (**Figure 4B**), there were chromosome arm gains in chromosome 7 and 5p in the majority of the samples and losses in 8p, 18q, and 21q. Recurrent gain of 7, 8q, and 5p and loss of 8p, 18, and 21 were also previously reported by Pickering et al. (21). **Figure 4B** also shows a Circos plot obtained from the PURPLE pipeline for CSCC_0004 as a representative example that summarizes various information at the sample level.

Structural variation analysis revealed that CSCC metastases are characterized by various complex, deleted, and unbalanced translocation events. **Table 2** provides the summary of various structural events observed. Deletion and complex structural variants are common in CSCC; however, unbalanced translocation and other structural events were also observed (**Table 2**). The detailed effects of these structural events for putative oncogenes and tumor suppressor genes (TSGs) are described in **Table 3**. Amplification events are linked to complex structural variants. Potential oncogene/TSG driver amplification and deletion were predicted by the PURPLE-GRIDSS-LINX pipeline, as reported in **Table 3**. Recurrent gene deletions were more common than gene amplifications. The most frequently deleted gene was *PTPRD (*Chr9p, 24% of samples). *PTPRD* deletion is already reported in primary and metastatic CSCC (44, 45). Deletion of *PTPRD* (n=6) and *CDKN2A* (Chr9p) (n=1) did not co-occur in our cohort (**Table 3**), although *PTPRD* loss and significant mutation of *CDKN2A* co*-*occurred in six samples (CSCC_9, 11, 12, 133, 132, and 134) (**Table 3**; **Figure 2G**). Deep deletion of *CDKN2A* was reported in only 2/92 cases available on cBioPortal (**Supplementary Figure S1**).

**Table 2:** Summary of various event categories of structural variants.

Sample	SGL	DEL	DUP	Complex	UNBAL_trans	Pair.other	INF
CSCC_0001	*SMAD4*	*SMAD4*					* *
CSCC_0002		*CDKN2A*					* *
CSCC_0005			*MYC*	*MYC*			* *
CSCC_0007				*CRLF2*			* *
CSCC_0009		*PTPRD*					* *
CSCC_0011		*PTPRD*		*CALR*	*HEBP2- NTRK2*		* *
CSCC_0012		*PTPRD*		*EGFR*		*PTPRD*	* *
CSCC_0013		*APC*					* *
CSCC_0014		*CREBBP*					*CREBBP*
CSCC_0025		*CDKN2C*			*PARD6G*		* *
CSCC_0066		*PTPN13*					* *
CSCC_0124		*NEGR1*				*NEGR1*	* *
CSCC_0132		*PTPRD*		*RAF1-FGF3-CCND1*			
CSCC_0133	*PTPRD*	*PTPRD*		CALR-chr1-chr3-chr6-chr8-chr22			
CSCC_0134				*MCL1, CCND1-FGF3*-Chr17			
CSCC_0135		*PTPRD*					

For more details, refer to **Supplementary Figures S4** and **S5**. Association can be noted between gain (**Table 3**) and complex SV events. The gene list was derived using LINX output. Only samples with events are shown in the table.

NBAL_TRANS, unbalanced translocation; INF, inferred breakend; DEL, deletion; DUP, duplication; SGL, single breakend SV support

**Table 3:** List of reportable drivers (likelihood type onco/TSG) genes.

Sample	DEL	GAIN	LOH_CHR	LOH_ARM	LOH	LOH_SV_TELO	LOH_SV_CENTRO
CSCC_0001	*SMAD4*					*SMAD4*	* *
CSCC_0002	*CDKN2A*						* *
CSCC_0003	*KDM6A*		*KDM6A*				* *
CSCC_0005	* *	*MYC*					* *
CSCC_0007	* *	*CRLF2*					* *
CSCC_0009	*PTPRD*			*PTPRD*			* *
CSCC_0011	*PTPRD*	*CALR*		*PTPRD*			* *
CSCC_0012	*PTPRD*	*EGFR*	*PPP2R3B, PUDP, STS,WWC3*		*PTPRD*		* *
CSCC_0013	*APC*			*APC*			* *
CSCC_0014	*CREBBP*					*CREBBP*	* *
CSCC_0025	*CDKN2C, PARD6G*		*PARD6G*	*CDKN2C*			* *
CSCC_0066	*PTPN13*		*PTPN13*				* *
CSCC_0124	*NEGR1*				*NEGR1*		* *
CSCC_0132	*PTPRD*	*RAF1,CCND1,FGF3*		*PTPRD*			* *
CSCC_0133	*PTPRD*	*CALR*					*PTPRD*
CSCC_0134	* *	*MCL1,CCND1,FGF3*					* *
CSCC_0135	*PTPRD*	* *	* *	*PTPRD*	* *	* *	* *

The types of drivers are as follows: GAIN, amplification by SV; DEL, homozygous deletion; LOH, focal LOH; LOH_ARM, chromosome arm level LOH; LOH_CHR, chromosome level LOH; LOH_SV_TELO, LOH from SV to telomere; LOH_SV_CENTRO, LOH from SV to centromere. Only samples with events are shown in the table.

Loss of heterozygosity (LOH) was found at the focal, arm, chromosome, telomere, and centromere levels. The most common LOH events were that at the chromosome and arm level with these events concentrated to *PTPRD* locus (**Table 3**). No recurrent events for other genes were observed (**Table 3**). Various examples of *PTPRD* structural events are reported in **Supplementary Figure S4**. A few other examples of the unbalanced translocation and complex structural variants are shown in **Supplementary Figure S5**.

The most frequently amplified genes (2/25, 8%) were *CALR*, *CCND1*, and *FGF3* (**Table 3**). Interestingly, *EGFR* was amplified in only one sample. Amplification of *CCDN1* and *FGF3* co-occurred in two samples (CSCC_0134 and CSCC_0132). *CCDN1* and *FGF3* are next to each other on the chromosome. These two cases had extensive nodal involvement (>50% of lymph nodes harboring tumor).

Despite this widespread genomic instability, only two coding–coding gene fusions were observed in our cohort. The first was between *STRN* and *DLG2* in sample CSCC_0009 (*STRN*: exon 1 ENST00000263918; *DLG2*: exon 7 ENST00000376104). *STRN* encodes a calcium-dependent calmodulin-binding protein (46). *DLG2* plays a role in pain signaling, and deletion is seen in both human and canine osteosarcoma (47). We noted above that CSCC_0009 is the only sample without *TP53* mutations. CSCC_0009 came from a patient who had undergone liver transplantation and was on immunosuppressive therapy. The primary tumor that gave rise to this metastasis showed perineural involvement, which was also present in the metastatic deposit. The second gene fusion was between *NTRK2* and *HEBP2* in CSCC_0011. This seems to be caused by an unbalanced translocation event (**Supplementary Figure S5B**).

### Enrichment analysis

**Figure 5:**
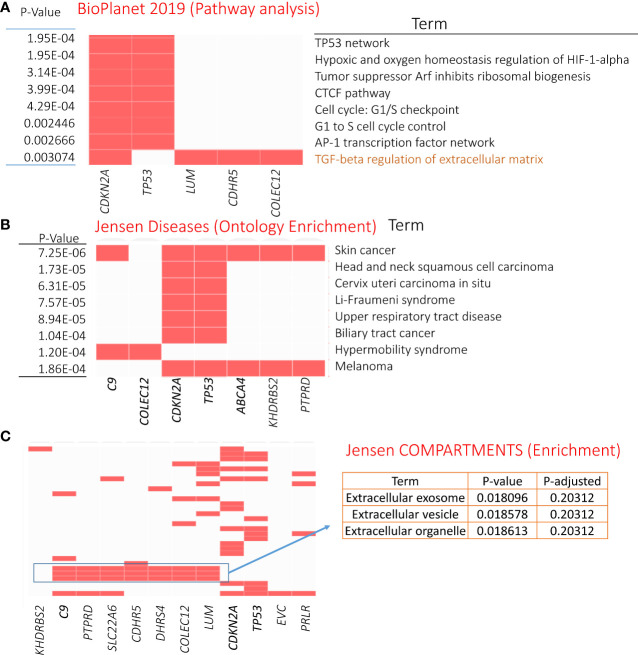
Enrichment analysis results of genetically mutated genes (21 candidates). **(A)** GO-Cellular Component terms showing eight significantly enriched terms (obtained from BioPlanet 2019). Panels **(B, C)** showing most significant Jensen diseases and Jensen compartments enriched terms, respectively. For details, refer to **Supplementary Table S6**.

Gene enrichment analysis was performed using the 21 genetically altered candidates identified above as significant/candidate driver genes, i.e., *TP53*, *CDKN2A*, *C9*, *KHDRBS2*, *SLC22A6*, *COLEC12*, *LINGO2*, *CDHR5*, *ZNF442*, *C9orf131*, *PRLR*, *DHRS4*, *PPP1R1A*, *EVC*, *LUM*, *ABCA4*, *LINC01003*, *LINC01474 (RP11-151D14.1)*, *RP4-597N16.4*, *RP11-61J19.4*, and *PTPRD*. The top significant pathway enrichment terms [Bio Planet 2019 (48)] are shown in **Figure 5A**. Most of the significant BioPlanet-enriched terms come from *TP53* and *CDKN2A*, such as TP53 network, tumor suppressor ARF, CTCF pathway, and cell cycle (G1/S checkpoint). However, *CDKN2A*, *LUM*, *CDHR5*, and *COLEC12* contribute to important cancer-related enrichment pathways, such as “TGF-beta regulation of extracellular matrix.” Full details of these enrichment analyses are available in **Supplementary Table S6**.

The Jensen diseases enrichment tool identified skin cancer with highest significance (**Figure 5B**), with Jensen compartment-based enrichment analysis showing that most of these genes belong to the extracellular compartment (**Figure 5C**). Other ontology enrichment analysis (MGI mammalian phenotype level 4 2021; **Supplementary Table S6**) showed enrichment of increased fibroblast proliferation MP:0011703 where *CDKN2A*, *TP53*, and *LUM* alterations are the main contributors.

**Figure 6:**
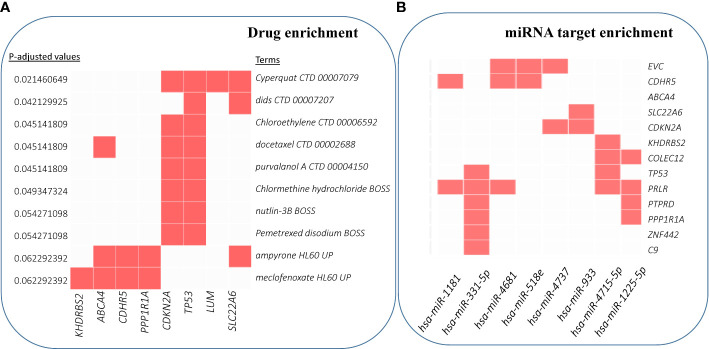
Enrichment analysis results for drug and miRNA targets. **(A)** Over-enrichment analysis of 20 driver candidates (deleted *PTPRD* excluded) against DSigDB (Drug SIGnatures DataBase) (49) annotation showing top 10 significantly enriched Drug/Compound. **(B)** Computationally predicted targets of miRNAs (TargetScan miRNA 2017). The x-axis represents the significance of the term (decreasing from left to right). (For details, refer to **Supplementary Table S6**).

We also performed enrichment analyses to predict drugs and miRNA targets for these driver candidates. **Figure 6A** shows the top 10 significant hits against drug annotations, which suggests that many of these driver genes are known therapeutic targets (dSig; **Supplementary Table S6**). With respect to miRNA targets, *hsa-miR-331-5p* was predicted to interact with six driver gene candidates, including *TP53* and *C9* (**Figure 6B**). For this prediction, the enricher platform uses TargetScan miRNA database (50). At the same time, *hsa-miR-1181* was one of the most significantly enriched miRNAs for these driver candidates, but can target only two driver genes.

## Discussion

This is the largest study to employ WGS to assess the mutational landscape of metastatic CSCC and demonstrates the breadth of somatic variation across non-coding and coding regions. Furthermore, we updated and expanded the understanding of UV-mutational signature patterns in metastatic CSCC (12), including the identification of novel indel (ID) signature patterns. This highlights for the first time the nature and depth of variation within regulatory regions, with special attention devoted to UTR and lncRNA. Additionally, we reported various structural events at whole genome scale for this diseases and also compared driver genes and SNVs to previous WES/targeted NGS studies on metastasis CSCC.

At 238 mutations/Mb (median of 166.99 mutations/Mb) within metastatic CSCC at the whole genome scale, the rate of TMB is substantially higher than that of other cancers known to have a high mutational burden, including melanoma, which is 49 mutations/Mb (51). Pickering et al. (21) found a median of 61.2 mutations/Mb from their WES of high-risk primary (n= 32) and metastatic (n =7) CSCC. Their finding shows lower TMB than our study because they analyzed only coding DNA, which has much lower TMB than non-coding DNA in CSCC (12). The high TMB was associated with substantial structural variation, without recurrent gene fusions.

**Figure 7:**
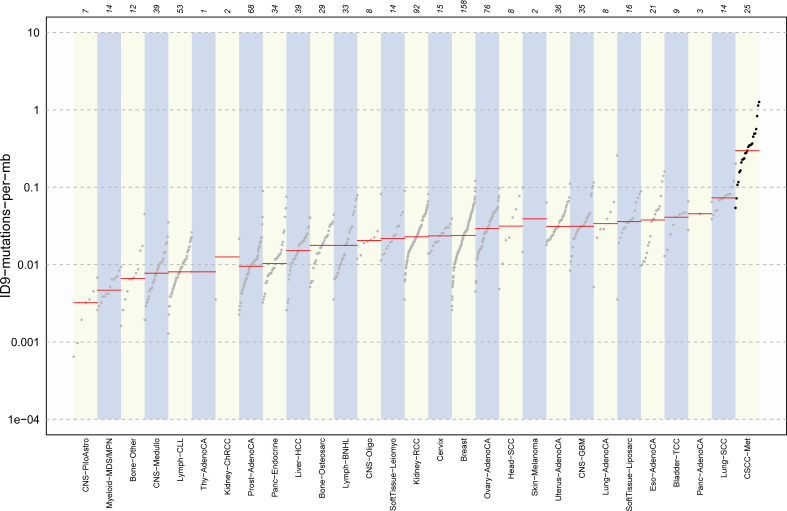
Comparison plot of ID9 mutations for various cancers. CSCC shows the highest ID9 mutations per Mb. The bottom x-axis represents the cancer types, and the upper x-axis shows the number of samples measured for specific cancer types. y-Axis indicates the number of mutations per Mb. Data for other cancers was obtained from ID9 signature details from COSMIC V3.2 and compared with CSCC data. CSCC data is calculated as ID9 signature score/3100 (coverage for hg38 genome).

Alexandrov et al. (52) detailed patterns of mutational signatures in 23,829 tumor samples (1,965 WGS) from the Pan Cancer Analysis of Whole Genomes (PCAWG) datasets including 17 small ID signatures, expanded to 18 in COSMIC version 3.2 (https://cancer.sanger.ac.uk) (53). However, no cutaneous SCCs (primary or metastatic) are included in this dataset. We identified the predominance of ID signatures 8, 9, and 13 (100% of samples effected) in our 25 metastatic CSCC samples. ID8 is thought to be both related to double-strand DNA break repair dysfunction and to age-related changes. Melanoma is the only other cancer type reported to have a predominant ID 13 signature (52). Our data also provide evidence of concomitance of ID13 with SBS 7a and 7b (**Figures 1C, D**; **Supplementary Table S2**) in keeping with a UV-mediated mechanism for this signature. While we found ID9 to be a dominant indel signature in CSCC, it is rare in melanoma (2/104) but predominant in soft tissue sarcoma (52). The mechanism of ID9 is unclear, but this departure from what is found in melanoma clearly shows some point of difference in these UV-induced skin cancers. When comparing the TMB associated with ID9 signature among different cancers, the dominance in CSCC is clearly visible (**Figure 7**). One case of SBS32 is due to azathioprine exposure.

We identified substantial somatic variation within the 3′UTR region of *EVC*, *LUM*, and *PPP1R1A. EVC* affects ciliary Hedgehog (Hh) regulation. Aberrant overexpression of *EVC* (and upregulation of Hh) has been reported in adult T-cell leukemia as a result of epigenetic modulation (54). The expression of *EVC* is reduced in nodal deposits of metastatic breast cancer compared with primary breast cancer, suggesting a role in the metastatic process (55). *LUM* is a major keratan sulfate proteoglycan that plays a role in collagen fibril organization, circumferential growth, epithelial cell migration, and tissue repair, among many other functions (56). *PPP1R1A* encodes a protein phosphatase inhibitor, which appears to have a variable but significant role in the metastatic process. For example, it is overexpressed in Ewing sarcoma and has been proposed as a driver of metastasis (57). Conversely, levels of *PPP1R1A* were reduced in breast cancer when compared to adjacent non-diseased breast tissue (58). Within our cohort, we observed a unique recurrent missense mutation in the 3′UTR of *PPP1R1A* in five samples.

*LINC01003* was the most mutated lncRNA in our cohort (64% of samples). In multiple myeloma, *LINC01003* behaves as a tumor suppressor genomic element. Upregulation suppresses multiple myeloma by repressing cell viability and adhesion and promoting apoptosis. This effect is *via* its sponge effect on miR-33a-5p and its target *PIM1* (59).

As has been frequently reported for CSCC (5) (**Supplementary Figure S1**), *TP53* and *CDKN2A* were also the most recurrently altered genes in our cohort. Loss of function mutations within *TP53* and *CDKN2A* are well known to adversely impact cell cycle pathway control and DNA repair mechanisms, thus increasing TMB. Furthermore, *TP53* and *CDKN2A* mutations in other squamous cell carcinomas such as NSCLC (60) and HNSCC (61) correlates with response to immune checkpoint inhibitors. With *TP53* and *CDKN2A* as driver genes in our study, the generally high response rates to immune checkpoint inhibitors in advanced and metastatic CSCC is not surprising. Kilnakis et al. (62) describe a pattern of *TP53* mutation that differed between primary and metastatic disease in head and neck (mucosal) SCC. They found an overall lower rate of mutations in metastatic tumors but a higher concentration of missense mutations in the DNA binding regions of the gene. However, Yilmaz et al. (17) reported a significantly higher *TP53* mutation frequency in metastatic (85%) compared to primary CSCC (corrected p-value <0.002). Our cBioPortal dataset analysis indicated no difference in variant frequency for *TP53* between primary and metastatic CSCC (refer to **Supplementary Figure S1**), suggesting retention in metastatic tumors.

Of note in our study was the absence of significant or recurrent SNVs affecting *NOTCH1/2*. Inman et al. (15) compared well-differentiated to moderately and poorly differentiated primary CSCC and identified *NOTCH1*, *NOTCH2*, *TP53*, and *CDKN2A* as the most commonly mutated genes, with *ATP1A1*, *HERC6*, *MAPK1P1L*, *GRHL2*, *TRAPPC9*, *FLNB*, and *MAP3K9* identified as common early events in primary CSCC. Within this group, *GRHL2* was associated with less well-differentiated tumors including those with a worse prognosis. In our cohort, only a single splice variant in *GRHL2* was identified, suggesting that its role in metastatic disease is limited.

*C9* (encodes complement component 9) was also identified as a potential driver gene by three driver identification tools, with SNVs identified in 52% of the samples in our cohort. C9 is part of the membrane attack complex (MAC) and has been shown to modulate cellular behavior in the tumor microenvironment (TME) (63). Since the TME plays a crucial role in tumorigenesis, progression, metastasis, and recurrence, C9 might have significant potential in CSCC progression to metastasis. Various other components of the complement system have been linked to CSCC progression and immunosuppression and implicated as potential therapeutic targets (64–66). With respect to *C9* specifically, it appears to be recurrently mutated in CSCC specimens (31% in primary and 10% in metastatic CSCC) as identified in the cBioPortal database (**Supplementary Figure S1**). and high expression levels have been proposed as a potential biomarker for the detection of gastric cancers (67) (68). Furthermore, the restrained expression of *C9* in tumor-associated macrophages promotes non-small cell lung cancer progression (69).

Apart from *TP53*, *CDKN2A*, and *C9*, we identified nine other potential driver genes with the most recurrently mutated gene being *KHDRBS2* (48% of cohort) with various impacts, including stop gained, complex, and synonymous types apart from missense variant across the cohort. In the cBioPortal database, this gene is mutated in 20% of metastatic CSCC specimens (**Supplementary Figure S1**), suggesting that it is a reasonably recurrently mutated gene in this disease.

A comparison of mutational frequency of primary and metastatic CSCC on the cBioPortal data suggests the potential of *COLEC12* (primary=25%; metastatic=60%) and *SLC22A6* (primary=16%; metastatic=30%) as drivers in metastatic CSCC (**Supplementary Figure S1**). Both *COLEC12* and *SLC33A6* are mutated in 44% of the samples in our cohort, and many of them are high-impact SNVs. *COLEC12* is involved in leukocyte recruitment and cancer metastasis (70) and regulates the apoptosis of osteosarcoma (70). Moreover, *COLEC12* is a potential biomarker of anaplastic thyroid cancer (ATC) (71). In one study of cancerous gastric stromal cells (GSCs), the role of *COLEC12* is found in mediating the crosstalk between GSCs and dendritic cells (DCs) (72). On the other hand, *SLC22A6* is known as an organic anion transporter 1 (*OAT1*). Expression and function alterations of *OAT1* play an essential role in therapeutic efficacy and the toxicity of many drugs, such as for anti-cancer drugs methotrexate, bleomycin, and cisplatin-related toxicity (73–75). *OAT1* variation associated with cardiotoxicity in pediatric acute lymphoblastic leukemia and osteosarcoma (76). Furthermore, the role of *OAT1* in breast cancer metastasis has been reported (77). Important cancer-related roles of the other potential CSCC drivers are reported in **Supplementary Table S7**.

Loss of *PTPRD* was the most prominent copy number alteration in our 25 samples. *PTPRD* encodes protein tyrosine phosphatase receptor D, which belongs to a family of receptors whose action opposes that of the tyrosine kinases, which are central to cell growth and differentiation and oncogenic transformation. Large-scale genomic events impacting *CDKN2A* can also affect *PTPRD* due to their proximity on chr9 (78). In head and neck SCC, *PTPRD* inactivation significantly increases *STAT3* hyperactivation, which was associated with decreased survival and resistance to epidermal growth factor receptor (EGFR)-targeted therapy (79). *PTPRD* has been implicated as a tumor suppressor in several cancers with inactivating somatic variants found in >50% of GBM and between 10% and 20% of head and neck mucosal SCC (HNSCC) (80). Lambert et al. (45) described deletions of *PTPRD* in 37% of metastatic primary CSCC and metastases. In addition, some of their cases also displayed a variant in the minor allele concordant with the deletion leading to a LOH event. It is thus possible that *PTPRD* plays a tumor suppressor role in preventing metastatic CSCC.

There were no recurrently amplified genes except for *CALR*, *CCND1*, and *FGF3*, which were each only amplified in 2/25 samples (**Table 3**). *CALR* encodes a ubiquitous endoplasmic-reticulum-bound calcium receptor (81). Cellular stress can move *CALR* fragments to the plasma membrane from the ER and influence immune recognition of cancer cells. Recent analysis of *CALR* fragments in myeloproliferative disease suggests an immunosuppressive influence of extracellular *CALR* (82). Cyclin D1 (*CCND1*) amplification is associated with nodal metastasis and worse survival in oral SCC (83). In a review of *CCND1* copy number variation in metastatic non-cutaneous melanoma, amplification was prominent in those patients whose disease did not respond to immune checkpoint inhibition (84). *FGF3* amplification is more common in metastatic breast cancer than primary tumors (85). Targetable *FGF3* amplification was associated with a poorer prognosis and lung metastasis in hepatocellular carcinoma (86). This amplification was seen in only 2% of total HCC but was most common in those cancers showing rapid response to sorafenib.

With respect to enrichment of driver gene alterations observed in our samples, dysregulation of the cell cycle pathway appears to be the central genomic theme of metastatic CSCC supported mainly by *TP53* and *CDKN2A*. *CDKN2A* encodes the CDK inhibitor p16^INK4a^. This inhibitor is an important controller of the activity of CDKs and progression from G1 to mitosis in the cell cycle. Inactivating mutations in *CDKN2A* with effects on p16^INK4a^ regulatory functions uncouple cell cycle control to promote cell survival and tumorigenesis (87). CDK4/6 inhibitors such as palbociclib, which has demonstrated response in metastatic breast cancer, may likewise be a potential therapeutic strategy for metastatic CSCC. Interaction between *CDKN2A* and *TP53* through *MDM2* and its regulation by ARF (also encoded by *CDKN2A*) further disable cell cycle and apoptotic pathways (GO: molecular function enrichment shows MDM2/MDM4 family protein binding). The pro-tumorigenic functions of the p53-MDM2-ARF network is gaining traction as a target for novel therapeutic strategies (88), which could also be applied to CSCC.

The cellular process defined by the term “TGF beta regulation of extra cellular matrix” was also significantly enriched showing a role for *LUM*, *CDHR5*, *COLEC12*, and *CDKN2A* in this process (**Figure 5A**). Compartment enrichment analysis found that these genetically altered genes are part of the extracellular compartment. Our previous differential expression study confirmed that TGFβ and the extracellular matrix component have an important role in metastatic CSCC (89). Inactivation of cell cycle control (through *CDKN2A* alterations for example) would allow tumor cells to escape from TGFβ-mediated suppressive effects. As loss of this growth-inhibitory response occurs at a level downstream of the core TGFβ signaling pathway, TGFβ then switches to a tumor-progression factor promoting epithelial-to-mesenchymal transition while inhibiting proliferation, differentiation, and the antitumor activity of multiple immune cells (90). As TGFβ receptor inhibition in combination with gemcitabine or immunotherapy is showing promise in other cancers (91, 92), this approach may also be applicable to metastatic CSCC.

Finally, *miR-331-5p* shows promise as a potentiator of CSCC drivers. *miR-331-5p* downregulation contributes to chemotherapy resistance/relapse in leukemia (93), and it inhibits proliferation by targeting PI3K/Akt and ERK1/2 pathways in colorectal cancer (94).

## Conclusion

WGS provides insight into the unparalleled burden of mutation within metastatic CSCC, and our study has provided a deeper understanding of the genomic complexity of this disease. The functional impact of the varied and complex genetic alterations observed in metastatic CSCC should be validated in the future in confirmatory studies comparing whole genomes of non-metastatic primary tumors to metastatic tumors. This knowledge would significantly contribute to the identification of biomarkers in primary CSCC for predicting metastasis.

## Data availability statement

The original contributions presented in the study are included in the article/**Supplementary Materials**. The variant call format files have been deposited at the European Genome-Phenome Archive, which is hosted by the EMBL-European Bioinformatics Institute and the Center for Genomic Regulation, under accession number EGAS00001006378.

## Ethics statement

This study was undertaken with Institutional Human Research Ethics approval (UOW/ISLHD HREC14/397). The patients/participants provided their written informed consent to participate in this study.

## Author contributions

AT and DS performed the bioinformatics analyses. BA and NGI conceived the idea and assisted in bioinformatics analyses. BA, AT, and MR drafted manuscript versions. BA, MR, RG, and JC obtained funding for the project. BA, JC, JM, SM, SC, and RG collated samples and/or clinical data. JP and EM completed tissue processing. All authors reviewed and edited the manuscript. All authors contributed to the article and approved the submitted version.

## Funding

This work was funded by the Illawarra Cancer Carers, The Head and Neck Research Fund, Royal Prince Alfred Institute of Academic Surgery, The Cancer Institute NSW translational program grant, Chris O’Brien Lifehouse, National Health and Medical Research Council Project Grant APP1181179, and Tour de Cure. The authors would like to acknowledge A/Prof Carsten Palme and Dr. Kerwin Shannon for suggestions and National Computational Infrastructure (NCI-GADI) and Sydney Informatics Hub for computational services.

## Conflict of interest

The authors declare that the research was conducted in the absence of any commercial or financial relationships that could be construed as a potential conflict of interest.

## Publisher’s note

All claims expressed in this article are solely those of the authors and do not necessarily represent those of their affiliated organizations, or those of the publisher, the editors and the reviewers. Any product that may be evaluated in this article, or claim that may be made by its manufacturer, is not guaranteed or endorsed by the publisher.

## Footnotes

1 Staging according to AJCC 8th edition. 

2 Lymph node ratio (Number of positive nodes/total nodes harvested).

3 Grade 1: well differentiated; Grade 2: moderately differentiated; Grade 3: poorly differentiated.
