# Supplementary material for: Whole genome analysis reveals the genomic complexity in metastatic cutaneous squamous cell carcinoma
Source: Front Oncol. 2022 Aug 2;12:919118. doi: 10.3389/fonc.2022.919118 (PMC9379253; doi:10.3389/fonc.2022.919118)
Supplement: Supplementary file 9 [file Table_7.docx]

| **Gene** | **Role** | **Associated with** | **References** |
| --- | --- | --- | --- |
| KHDRBS2 | Carcinogenesis, alternating splicing in cancer | Aggressive renal cell carcinoma's, stage 4 neuroblastoma, survival in lung adenocarcinoma and Glioblastoma, papillary thyroid carcinomas | [1-5] |
| LINGO2 | Polymorphisms in the 9p21 region (*LINGO2*) are associated with the risk of multiple cancers, cellular proliferation and the development of features common to cancer cells. | Survival in advanced gastric cancers, interaction of LncRNA *LINC00518* and *LINGO2* acts as an Oncogene in Uveal Melanoma | [6-9] |
| CDHR5 | Encodes cadherin-related family member 5 | Prognostic marker of progression in clear cell renal cell carcinoma, inhibits proliferation of hepatocellular carcinoma, promotes malignant phenotype of pancreatic ductal adenocarcinoma | [10-12] |
| *ZNF422* | Specific to ethnicity or the tumor's heterogeneous populations | lung cancer (epigenetically silenced), homeostasis of MCF-7 breast cancer cells | [13-15] |
| PRLR | Carcinogenesis, GH signaling, EGFR/ERBB signaling pathways | metastatic risk in Breast cancer, carcinogenesis in cervical, ovarian, and endometrial cancers, a potential target for cancer treatment in prostate and human breast cancer | [16-20] |
| *DHRS2* | p53 regulation, cell cycle, apoptosis, and drug resistance in carcinoma cells | Motility in esophageal squamous cell carcinoma, poor prognosis in ovarian cancer, and a potential marker of breast cancer metastasis | [21-23] |

**Supplementary** **Table 7.** **Known** **role of other potential cSCC drivers in cancer.**

**References:-**

1. Inamura, K., *Translocation Renal Cell Carcinoma: An Update on Clinicopathological and Molecular Features.* Cancers (Basel), 2017. **9**(9).

2. Bell, J.L., et al., *Identification of RNA-Binding Proteins as Targetable Putative Oncogenes in Neuroblastoma.* Int J Mol Sci, 2020. **21**(14).

3. Hou, Z., et al., *A Potential Prognostic Gene Signature for Predicting Survival for Glioblastoma Patients.* Biomed Res Int, 2019. **2019**: p. 9506461.

4. Li, W., et al., *Integrated analysis of the roles and prognostic value of RNA binding proteins in lung adenocarcinoma.* PeerJ, 2020. **8**: p. e8509.

5. Passon, N., et al., *Somatic amplifications and deletions in genome of papillary thyroid carcinomas.* Endocrine, 2015. **50**(2): p. 453-64.

6. Jo, J.H., et al., *Novel Gastric Cancer Stem Cell-Related Marker LINGO2 Is Associated with Cancer Cell Phenotype and Patient Outcome.* Int J Mol Sci, 2019. **20**(3).

7. Benisty, H., et al., *Mutation bias within oncogene families is related to proliferation-specific codon usage.* Proc Natl Acad Sci U S A, 2020. **117**(48): p. 30848-30856.

8. Barbagallo, C., et al., *LncRNA LINC00518 Acts as an Oncogene in Uveal Melanoma by Regulating an RNA-Based Network.* Cancers (Basel), 2020. **12**(12).

9. Li, W.Q., et al., *Genetic polymorphisms in the 9p21 region associated with risk of multiple cancers.* Carcinogenesis, 2014. **35**(12): p. 2698-705.

10. Bläsius, F.M., et al., *Loss of cadherin related family member 5 (CDHR5) expression in clear cell renal cell carcinoma is a prognostic marker of disease progression.* Oncotarget, 2017. **8**(43): p. 75076-75086.

11. Ding, X., et al., *CDHR5 inhibits proliferation of hepatocellular carcinoma and predicts clinical prognosis.* Ir J Med Sci, 2020. **189**(2): p. 439-447.

12. Gao, J., et al., *Up-regulation of CDHR5 expression promotes malignant phenotype of pancreatic ductal adenocarcinoma.* J Cell Mol Med, 2020. **24**(21): p. 12726-12735.

13. Zaman, M.S., et al., *Transcriptomic insights into the zinc homeostasis of MCF-7 breast cancer cells via next-generation RNA sequencing.* Metallomics, 2021. **13**(6).

14. Brim, H., et al., *Genomic aberrations in an African American colorectal cancer cohort reveals a MSI-specific profile and chromosome X amplification in male patients.* PLoS One, 2012. **7**(8): p. e40392.

15. Subramanian, D.N., et al., *Exome sequencing of familial high-grade serous ovarian carcinoma reveals heterogeneity for rare candidate susceptibility genes.* Nat Commun, 2020. **11**(1): p. 1640.

16. Shemanko, C.S., *Prolactin receptor in breast cancer: marker for metastatic risk.* J Mol Endocrinol, 2016. **57**(4): p. R153-r165.

17. Zhou, Y., et al., *A novel bispecific antibody targeting CD3 and prolactin receptor (PRLR) against PRLR-expression breast cancer.* J Exp Clin Cancer Res, 2020. **39**(1): p. 87.

18. Xu, J., et al., *The role of prolactin receptor in GH signaling in breast cancer cells.* Mol Endocrinol, 2013. **27**(2): p. 266-79.

19. Kavarthapu, R., R. Anbazhagan, and M.L. Dufau, *Crosstalk between PRLR and EGFR/HER2 Signaling Pathways in Breast Cancer.* Cancers (Basel), 2021. **13**(18).

20. Ramírez-de-Arellano, A., et al., *The Relevant Participation of Prolactin in the Genesis and Progression of Gynecological Cancers.* Front Endocrinol (Lausanne), 2021. **12**: p. 747810.

21. Zhou, Y., et al., *DHRS2 inhibits cell growth and motility in esophageal squamous cell carcinoma.* Oncogene, 2018. **37**(8): p. 1086-1094.

22. Han, Y., et al., *Decreased DHRS2 expression is associated with HDACi resistance and poor prognosis in ovarian cancer.* Epigenetics, 2020. **15**(1-2): p. 122-133.

23. Yaylaz, B.S., et al., *DHRS2 is a potential marker of breast cancer metastasis.* Gene Reports, 2021. **25**: p. 101302.
